# Unraveling the Biosynthesis of Carvacrol in Different Tissues of *Origanum vulgare*

**DOI:** 10.3390/ijms232113231

**Published:** 2022-10-30

**Authors:** Yuanpeng Hao, Xiaoqi Guo, Rui Yang, Yihao Yan, Meiyu Sun, Hui Li, Hongtong Bai, Hongxia Cui, Jingyi Li, Lei Shi

**Affiliations:** 1 Key Laboratory of Plant Resources, Institute of Botany, Chinese Academy of Sciences, Beijing 100093, China; 2 China National Botanical Garden, Beijing 100093, China; 3 University of Chinese Academy of Sciences, Beijing 100049, China; 4 Institute of Materia Medica, Chinese Academy of Medical Sciences & Peking Union Medical College, Beijing 100050, China

**Keywords:** *Origanum vulgare*, carvacrol, tissue dependence, SMRT, WGCNA

## Abstract

*Origanum vulgare*, belonging to the Lamiaceae family, is a principal culinary herb used worldwide which possesses great antioxidant and antibacterial properties corresponding to various volatile organic components (VOCs). However, the metabolite profiles and underlying biosynthesis mechanisms of elaborate tissues (stems, leaves, bracts, sepals, petals) of *Origanum vulgare* have seldom been reported. Here, solid-phase microextraction–gas chromatography/mass spectrometry results showed that *Origanum vulgare* ‘Hot and Spicy’ (*O. vulgare* ‘HS’) was extremely rich in carvacrol and had the tissue dependence characteristic. Moreover, a full-length transcriptome analysis revealed carvacrol biosynthesis and its tissue-specific expression patterns of ‘upstream’ MVA/MEP pathway genes and ‘downstream’ modifier genes of TPSs, CYPs, and SDRs. Furthermore, the systems biology method of modular organization analysis was applied to cluster 16,341 differently expressed genes into nine modules and to identify significant carvacrol- and peltate glandular trichome-correlated modules. In terms of these positive and negative modules, weighted gene co-expression network analysis results showed that carvacrol biosynthetic pathway genes are highly co-expressed with TF genes, such as *ZIPs* and *bHLHs*, indicating their involvement in regulating the biosynthesis of carvacrol. Our findings shed light on the tissue specificity of VOC accumulation in *O. vulgare* ‘HS’ and identified key candidate genes for carvacrol biosynthesis, which would allow metabolic engineering and breeding of *Origanum* cultivars.

## 1. Introduction

Throughout the plant world, numerous species generate aromatic essential oils (EOs) that include complex mixes of volatile organic compounds (VOCs), such as terpene or phenylpropanoid metabolites, which are of tremendous value in a variety of sectors, ranging from food chemistry to pharmaceutics [1]. Oregano belongs to the Lamiaceae family and is a well-known herb that is widely distributed in the Asia and the Mediterranean which can be used to extract an EO mainly dominated by compositions of phenolic monoterpenes (carvacrol, thymol) and their precursors- [2,3]. Despite the fact that the plant contains several chemotypes, the carvacrol chemotype (containing up to 80% carvacrol) is the most valuable due to its therapeutic activity and flavoring characteristics. Carvacrol (C_10_H_14_O) is a liquid phenolic monoterpenoid with antibacterial action and some pharmacological effects that have attracted interest [4,5]. Its use as a food additive is highly desirable because of its inclusion in the European Commission’s list of chemical flavorings and Food and Drug Administration (FDA) clearance as a toxicologically safe chemical (FDA, 2017).

The cytosolic mevalonate pathway (MVA) or the plastid-based methylerythritol pathway (MEP) are both viable routes for terpene production. Isopentenyl diphosphate (IPP) is generated in the cytosol by the condensation of two acetyl-CoA moieties via the MVA route. Starting with glyceraldehyde 3-phosphate (G3P) and pyruvate (PYR), the plastidic MEP pathway produces the isomer dimethylallyl diphosphate (DMAPP). Later, two C5 units fuse to produce geranyl diphosphate (GPP), a monoterpene precursor, and three C5 units fuse to form farnesyl diphosphate (FPP), a sesquiterpene precursor [6]. Typically, monoterpene synthases catalyze the cyclization of GPP via the α-terpinyl cation intermediate, and this very unstable intermediate can subsequently be transformed into certain monoterpenes [7]. Terpene biosynthesis pathways contain the terpene synthases (TPSs) and cytochrome P450-dependent monooxygenases (CYPs) as their central enzymes [8]. An important class of CYPs is responsible for the oxidation and conjugation of the first TPS products, which are involved in the modification of monoterpenes and sesquiterpenes [9]. In addition, a recent study demonstrated that the aromatic backbones of carvacrol are produced by the CYP71D subfamily and short-chain dehydrogenase/reductase (SDR) in conjunction with dehydrogenases via unstable intermediates [10].

Glandular trichomes are often multicellular and serve as the site of biosynthesis and storage of significant amounts of VOCs in a liquid state, which are only released when trichomes are broken [11]. Many plant species have glandular trichomes, and much literature on this topic has demonstrated the enormous variation in their shape and size [12]. The number of specific metabolites generated by a plant is frequently closely connected to the density of glandular trichomes present at the epidermis’s surface [13]. Increasing glandular trichome density is a novel plant breeding strategy for increasing beneficial chemical output.

The advent of short-read sequencing with Illumina technology and single-molecule real-time (SMRT) sequencing with the PacBio RS system has accelerated the creation of genome and transcriptome resources for various plant species [14,15]. Correlations between the expression of paired genes may be used to determine relationships based on transcript expression data, and a gene co-expression network can be built by discovering non-random gene–gene expression connections [16]. Hence, we explored the mechanisms underlying the generation of monoterpenes and sesquiterpenes in different elaborate tissues (stems, STs; leaves, LEs; bracts, BRs; sepals, SEs; petals, PEs) of *Origanum vulgare* ‘Hot and Spicy’ (*O. vulgare* ‘HS’) via the combined analysis of SMRT sequencing- and short-read sequencing-based gene expression patterns and solid-phase microextraction–gas chromatography/mass spectrometry (SPME-GC/MS)-based metabolite profiling. In addition, we performed a modular organization study of carvacrol biosynthesis-related genes and identified hub genes using the weighted gene co-expression network analysis (WGCNA) method.

## 2. Results

### 2.1. Volatile Organic Compound Profiles of O. vulgare ‘HS’ Captured by the SPME Fibre

**Figure 1:**
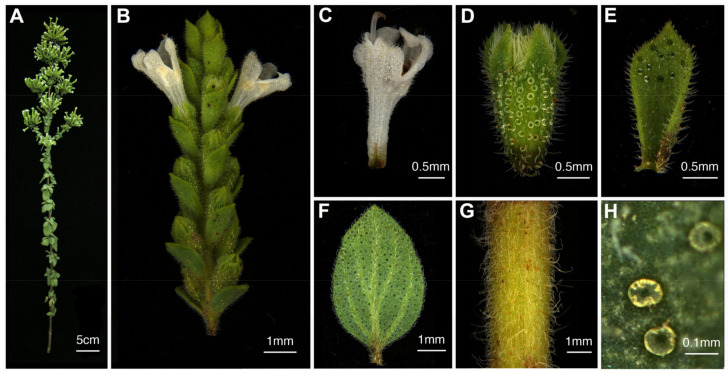
Photographs of *Origanum vulgare* ‘Hot and Spicy’ plants at the full bloom stage. The aerial part of *O. vulgare* ‘HS’ (**A**). The inflorescence of *O. vulgare* ‘HS’ (**B**). Different tissues of petals (PE; (**C**)), sepals (SE; (**D**)), bracts (BR; (**E**)), leaves (LE; (**F**)), and stems (ST; (**G**)). Peltate glandular trichomes of *O. vulgare* ‘HS’ (**H**).

For the elaborate flower structures of BRs, SEs, and PEs (Figure 1), we used SPME-GC/MS technology to detect the VOCs in aerial parts of *O. vulgare* ‘HS’ and found a total of 21 monoterpene and sesquiterpene compounds. Carvacrol was the major compound in LEs (84.71 ± 1.59%), BRs (96.07 ± 0.67%), SEs (96.92 ± 0.85%), and PEs (94.40 ± 1.23%) (Table S1). At the flowering stage, STs contained p-cymene at 65.44 ± 5.77% and carvacrol at 13.06 ± 6.74%.

**Figure 2:**
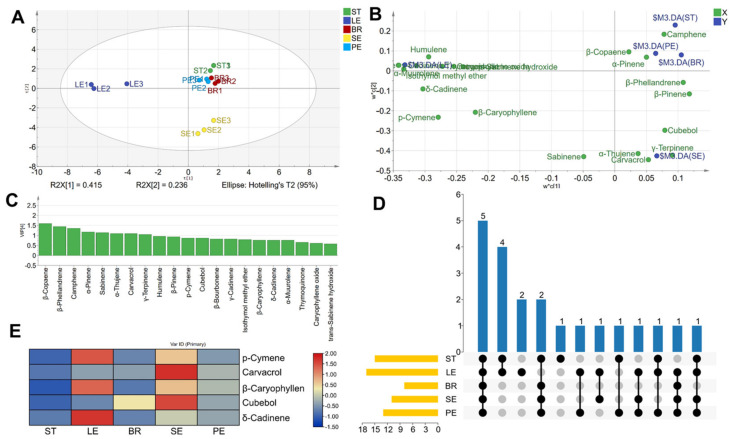
Analysis of volatile organic compounds from different tissues of *Origanum vulgare* ‘Hot and Spicy’. Score plot (**A**), loading plot (**B**), and variable importance in projection values (**C**) from PLS-DA model based on the chemical profiles of all samples. UpSet plot (**D**) and shared component Heatmap (**E**) based on the volatile organic compounds from different tissues of *Origanum vulgare* ‘Hot and Spicy’.

To conduct the quantitative analysis of chemical profiles in STs, LEs, BRs, SEs, and PEs of *O. vulgare* ‘HS’, we calculated the relative SPME peak area per unit mass. A supervised PLS-DA analysis approach was employed to differentiate tissue components (Figure 2A, Figure 2B). These results indicated that the volatile chemical compositions of different tissues had obvious differences. In addition, important volatile components were identified based on higher variable importance in the projection (VIP ≥ 1) values (Figure 2C). Moreover, shared and unique components of all samples were visualized by an UpSet analysis (Figure 2D). The number of chemical components present in different tissues ranged from eight to seventeen. Of these, five components were shared by all tissues, such as p-cymene, carvacrol, and β-caryophyllene, etc. Among these, SEs showed the highest carvacrol content, whereas STs lacked high amounts of components (Figure 2E).

### 2.2. SMRT Sequencing Achieves Full-Length Transcripts of O. vulgare ‘HS’

To obtain the precise and comprehensive full-length sequences of *O. vulgare* ‘HS’, we used the combination of SMRT and short-read sequencing technology via PacBio RS and Illumina platforms. First of all, we constructed two SMRT cells to obtain 21.84 Gb of clean data and acquired 308,935 circular consensus (CCS) reads (with the filter criteria of full passes ≥ 3 and sequence accuracy > 90%; Figure S1), and 270,324 full-length non-chimeric (FLNC) reads were identified (FLNC % = 87.50%; Figure S2). Accordingly, we used SMRTLink software to cluster these FLNC reads and achieved 101,447 consensus isoforms (Figure S3A). After polishing, we obtained 97,949 high-quality isoforms (sequence accuracy > 99%) and revised the low-quality isoforms using Proofread software compared to relevant Ilumina RNA sequence data. We merged all the high-quality and revised consensus isoforms, removed sequence redundancy through cDNA_Cupcake, and ultimately obtained 53,802 non-redundant sequences with an N50 of 2232 bp. Next, we used the MIcroSAtellite identification tool to analyze non-redundant sequences and certified 37,217 SSRs (Figure S4), and applied TransDecoder software to predict complete 40,365 CDSs and relevant protein sequences (Figure S3B; Table S2).

A total of 49,175 isoforms were annotated after analyzing the annotation, route, and functional categorization of SMRT data. Among them, 37,697, 49,049, 35,702, 21,634, 31,164, 40,288, and 21,462 isoforms were respectively annotated according to the GO (Gene Ontology; Figure S3C), NR (Non-Redundant; Figure S3D), Swissprot, COG (Clusters of Orthologous Groups; Figure S5), KOG (euKaryotic Ortholog Groups; Figure S6), Pfam (Protein family), and KEGG (Kyoto Encyclopedia of Genes and Genomes) databases. In detail, 37,697 isoforms annotated according to the GO database were classified into 3 main categories and distributed across 50 subcategories (Figure S3C). For the biological process (82,110 isoforms), the greatest number of isoforms were enriched in ‘metabolic process’ (20,210 isoforms; 24.61%), ‘cellular process’ (18,902 isoforms; 23.02%), and ‘single-organism process’ (14,270 isoforms; 17.38%). For the molecular function (45,571 isoforms), the greatest number of isoforms were enriched in ‘catalytic activity’ (20,561 isoforms; 45.12%) and ‘binding’ (18221 isoforms; 39.98%). As shown in Figure S3D, there were three major species (*Sesamum indicum*, 30,719 isoforms, 62.64%; *Erythranthe guttata*, 8640 isoforms, 17.62%; *Salvia miltiorrhiza*, 895 isoforms, 1.83%) with high similarity to *O. vulgare* ‘HS’. In this setting, SMRT technology was an efficient method for further biological study of plants and yielded a comprehensive transcriptome resource of *O. vulgare* ‘HS’.

### 2.3. RNA-Seq Data Reveal Carvacrol Biosynthesis in Different Tissues of O. vulgare ‘HS’

To identify the tissue-specific expression of key genes involved in carvacrol production in *O. vulgaris* ‘HS’ aerial parts, fifteen RNA samples from five different tissues with three biological replications of STs (ST1–3), LEs (LE1–3), BRs (BR1–3), SEs (SE1–3), and PEs (PE1–3) at the full bloom stage were subjected to short-read sequencing. Totally, we obtained 100.18 G clean data with a Q30 > 85% and an approximate GC content of 48.9% (Table S3). Moreover, we conducted the sequence alignment of short-read sequencing-based clean reads with the reference SMRT-based non-redundant transcripts, enabling us to achieve the expression patterns of SMRT transcripts (Table S4). To test the repeatability and reliability between sample biological replications, we observed the transcriptional abundance of each sample based on FPKM density and values (Figure S7A, Figure S7C), and conducted the Pearson Correlation Coefficient analysis of expression levels among samples (Figure S7B). Figure S7 indicates that the SE1 sample was extraordinary compared to SE2 and SE3. Biological replications of other PE, BR, LE, and ST samples clustered together. As a whole, we removed the SE1 sample and used the other 14 samples to carry out further gene differential expression analysis to obtain tissue-specific DEGs (FC > 2; FDR < 0.01; Table S5).

**Figure 3:**
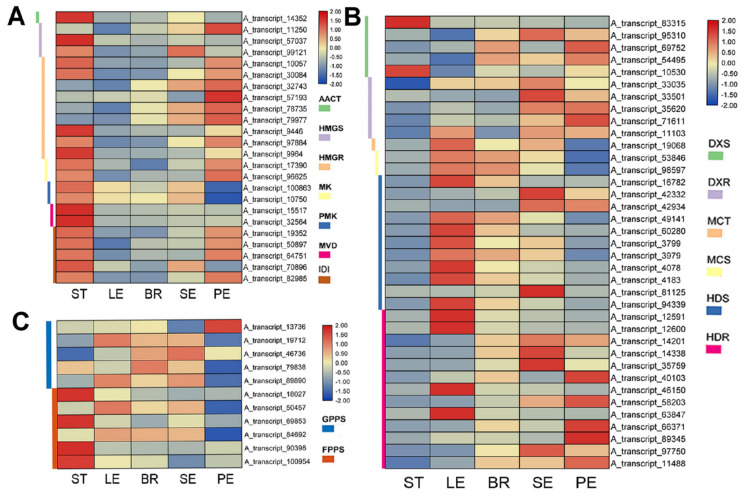
Transcript abundance profiles of genes encoding enzymes involved in the mevalonate (MVA) pathway (**A**), the methylerythritol pathway (MEP) pathway (**B**), and the biosynthesis of sesquiterpene skeleton (**C**) in different tissues of *Origanum vulgare* ‘Hot and Spicy’.

In this study, we predicated the biosynthesis pathways of monoterpenes and sesquiterpenes in *O. vulgare* ‘HS’, especially for carvacrol. As a result, we investigated the expression of DEGs encoding enzymes in different tissues. The DEGs were subjected to the filters of FC > 2 or < 0.5 and FDR < 0.01. Analysis of our transcriptome dataset revealed 24 (across 7 gene families) and 37 (across 6 gene families) candidate DEGs of *O. vulgare* ‘HS’ related to MVA and MEP pathways, respectively. As for MVA genes, 19 DEGs (79.17%) exhibited high expression levels in STs, 16 DEGs (66.67%) in SEs and PEs, 13 DEGs (54.17%) in BRs, and 10 DEGs (41.67%) in LEs (FPKM > 10), while STs presented more highly expressed DEGs than other tissues (FC > 2) (Figure 3A). Notably, five DEGs were most highly expressed in STs, comprising three IDIs (A_transcript_50897, A_transcript_19352, A_transcript_82985), one *AACT* (A_transcript_14352), and one *HMGR* (A_transcript_9446) (FPKM > 100). Two *IDI*s (A_transcript_19352, A_transcript_82985) were most highly expressed in PEs (FPKM > 100). As for MEP genes, 24 DEGs (64.86%) exhibited high expression levels in BRs, 23 DEGs (62.16%) in SEs, 21 DEGs (56.76%) in LEs, 16 DEGs (43.24%) in PEs, and 14 DEGs (37.84%) in STs (FPKM > 10) (Figure 3B). Compared to STs, 21, 23, 19, and 15 DEGs in SEs, BRs, LEs, and PEs presented upregulated expression (FC > 2). Significantly, three *HDR*s (A_transcript_14338, A_transcript_97750, A_transcript_35759) were most highly expressed in SEs, BRs, PEs, and one *HDS* (A_transcript_81125) in SEs (FPKM > 10; FC > 100). The gene expression patterns of five *GPPS*s and six *FPPS*s (Figure 3C) revealed that three of five *GPPS*s exhibited upregulated expression in SEs, LEs, and BRs (FC > 2) compared with STs, especially for *GPPS* (A_transcript_46736) highly expressed in SEs (FPKM = 38.02; FC > 100). Observably, *FPPS*s, contributing to sesquiterpene biosynthesis, presented more downregulations in PEs (6/6), SEs (4/6), BRs (4/6), and LEs (4/6) than in STs (FC < 0.5). In general, tissue-specific expression of MEP genes revealed maximum expression in SEs, followed by BRs, LEs, PEs, and STs. Conversely, MVA genes were highly expressed even in STs, followed by SEs, PEs, BRs, and LEs.

### 2.4. Transcriptomic Analysis of Tissue-Specific Expression Patterns of TPS/CYP/SDR Genes in O. vulgare ‘HS’

**Figure 4:**
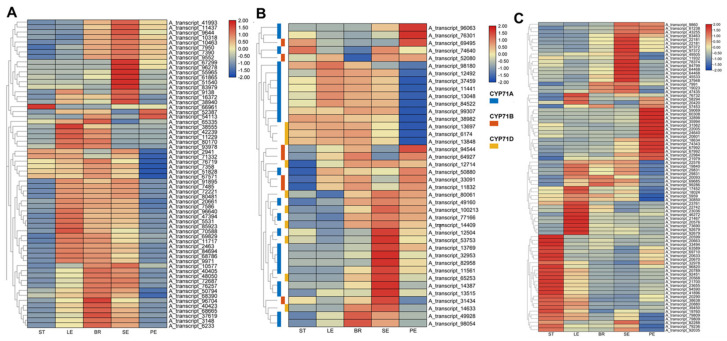
The expression pattern analysis of genes associated with terpene synthases (TPSs), cytochromes P450s (CYPs), and short-chain dehydrogenase/reductases (SDRs). Heatmap of expression profiles of TPS (**A**), CYP71 (**B**), and SDR (**C**) genes in different tissues of *Origanum vulgare* ‘Hot and Spicy’.

In *O. vulgare* ‘HS’, various distinct TPSs can be predicted for final products of monoterpenes and sesquiterpenes, especially for carvacrol content in SEs, PEs, LEs, and BRs, but not in STs. Importantly, γ-terpinene synthase gives rise to γ-terpinene, and other enzymes, such as CYPs and SDRs, are necessarily involved in later steps to confer final arrangements, generating the product of carvacrol [10] (Figure S8; red box). Other monoterpene synthases are implicated in forming various monoterpenes, such as sabinene. Based on the annotated database, we achieved 62 TPS DEGs and analyzed their expression patterns in STs, LEs, BRs, SEs, and PEs (Figure 4A). Of these, 17 SE-specific TPS genes were clustered and presented extremely low expression in STs. Significantly, three γ-terpinene synthase genes (A_transcript_9644, A_transcript_7390, A_transcript_11437) exhibited the highest expression in SEs and the lowest expression in STs.

An essential class of CYPs is responsible for the oxidation and conjugation of the initial terpene synthase products, and TPS genes are typically found in tandem with the CYP71 gene family in both eudicots and monocots [17]. In the present study, we analyzed 40 CYP71 family genes (including 23 *CYP71A*s, 7 *CYP71B*s, and 10 *CYP71D*s) in different tissues of *O. vulgare* ‘HS’ (Figure 4B). Compared with STs, 26 upregulated CYP genes in SEs contained 16 *CYP71A*s, 3 *CYP71B*s, and 7 *CYP71D*s; 16 upregulated CYP genes in BRs contained 9 *CYP71A*s, 4 *CYP71B*s, and 3 *CYP71D*s; 14 upregulated CYP genes in PEs contained 6 *CYP71A*s, 6 *CYP71B*s, and 2 *CYP71D*s; 12 upregulated CYP genes in LEs contained 8 *CYP71A*s, 3 *CYP71B*s, and one *CYP71D*. Simultaneously, 3, 4, 11, and 3 downregulated CYP genes were found in SEs, BRs, PEs, and LEs. Notably, five CYP genes (*CYP71A*s: A_transcript_14387, A_transcript_11561, A_transcript_12504, A_transcript_13769; *CYP71D*: A_transcript_14409) exhibited obviously higher expression in SEs than in other tissues (FPKM > 10). Next, we observed that seven *CYP71A*s and two *CYP71D*s were most highly expressed in SEs. These results indicate that CYP71 family genes prefer higher transcripts in carvacrol-rich tissues, especially for *CYP71As* and *CYP71D*s in *O. vulgare* ‘HS’.

A recent study has reported that γ-terpinene was converted to carvacrol by the combined activity of a CYP71D monooxygenase and the short-chain dehydrogenase/reductase (SDR) [10]. The subsequent formation of the aromatic compounds occurs via keto–enol tautomerisms. Based on the annotated database, we achieved 83 SDRs and analyzed their expression patterns in STs, LEs, BRs, SEs, and PEs (Figure 4C). Of these, 19 SE-specific (e.g., A_transcript_11932 and A_transcript_19023), 20 LE-specific (e.g., A_transcript_22742 and A_transcript_23036), and 15 PE-specific (e.g., A_transcript_74343 and A_transcript_31562) SDR genes were clustered and presented extremely low expression in STs.

### 2.5. Identification of VOC-Related Gene Modules Using Modular Organization Analysis

**Figure 5:**
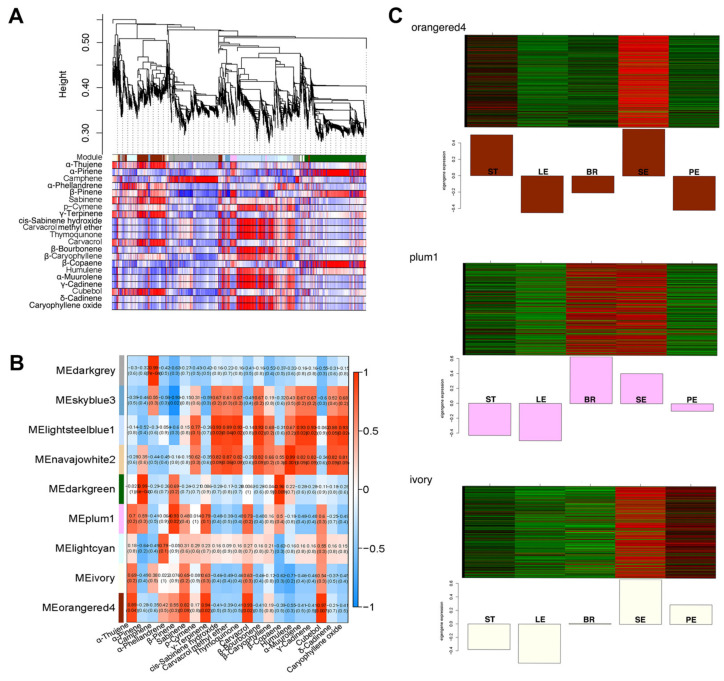
Modular organization analysis of gene expression in different tissues of *Origanum vulgare* ‘Hot and Spicy’ associated with volatile organic compounds (VOCs). The clustering tree depicts nine modules of co-expressed genes (**A**). Each of the 16,341 DEGs is represented by a leaf on the tree, and the primary branches of the tree represent modules, which are identified in different colors in the lower panel. The correlations between 21 VOCs and 9 modules (**B**). The expression profiles in different tissues of DEGs in orangered4, plum1, and ivory modules (positive with carvacrol content) (**C**).

In Figure S9A, using 16,341 DEGs generated from *O. vulgare* ‘HS’, a network heat map plot (interconnectivity plot) of the gene network is displayed, along with the related hierarchical clustering dendrograms and subsequent modules. Using a set of criteria that resulted in improved clusters, we identified nine modules with between 147 and 3,873 DEGs each, colored darkgrey, skyblue3, lightsteelblue1, navajowhite2, darkgreen, plum1, lightcyan, ivory, and orangered4 (Figure S9B). We also calculated the similarity and clustered nine modules into three subgroups depending on their eigenvalues: (i) the darkgrey, skyblue3, and lightsteelblue1 modules; (ii) the navajowhite2, darkgreen, and plum1 modules; (iii) the lightcyan, ivory, and orangered4 modules. To capture comprehensive transcriptome changes in different tissues of *O. vulgare* ‘HS’, we conducted modular organization analysis to classify 16,341 DEGs related to VOCs. The module–VOC relationship revealed that each component was significantly relevant to at least one module (R > 0.5; Figure 5A, Figure 5B). A large portion of carvacrol existed in SEs, BRs, PEs, and LEs, but not in STs, exhibiting a highly positive correlation with orangered4 (R = 0.93), plum1 (R = 0.73), and ivory (R = 0.63) modules. The vital intermediate product of γ-terpinene also presented a similar positive correlation with orangered4 (R = 0.94), plum1 (R = 0.79), and ivory (R = 0.63) modules. STs with a high content of p-cymene exhibited a strongly positive correlation with lightsteelblue (R = 0.77) and navajowhite (R = 0.62) modules (Figure 5B). Additionally, the expression profiles of DEGs in the orangered4 module appeared to be extremely elevated in SEs and STs, and DEGs in the plum1 module exhibited more upregulations in BRs and SEs, while DEGs in the ivory module presented more upregulations in SEs and PEs (Figure 5C).

With a highly positive correlation between orangered4, plum1, ivory module genes and carvacrol content, we conducted a GO and KEGG term analysis of these module DEGs (Figure S10). As for GO terms, DEGs enriched in ‘metabolic process’ and ‘cellular process’ showed a high percentage for biological process; DEGs enriched in ‘catalytic activity’ and ‘binding’ showed a high percentage for the molecular function; DEGs enriched in ‘cell part’ and ‘cell’ showed a high percentage for the cellular component (Figure S10A, Figure S10C, Figure S10E). As for KEGG terms, major DEGs were involved in the metabolism processes of ‘starch and sucrose metabolism’, ‘carbon metabolism’, ‘amino sugar and nucleotide sugar metabolism’, ‘biosynthesis of amino acids’, ‘glycolysis/gluconeogenesis’, and ‘pyruvate metabolism’ (Figure S10B, Figure S10D, Figure S10F). Notably, the processes of ‘starch and sucrose metabolism’, ‘glycolysis/gluconeogenesis’, ‘pyruvate metabolism’, and ‘carbon metabolism’ took part in generating G3P and PYR as the precursors of MVA and MEP pathways, and distinct module genes were differently expressed in these processes.

### 2.6. Modular Organization Analysis of Gene Expression Associated with Peltate Glandular Trichomes

**Figure 6:**
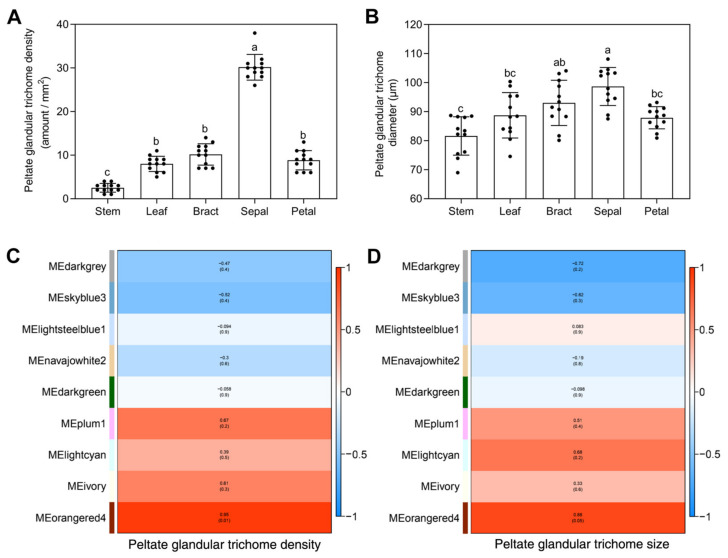
Modular organization analysis of gene expression in different tissues of *Origanum vulgare* ‘Hot and Spicy’ related to peltate glandular trichomes (PGTs). The density of PGTs in different tissues (**A**). The diameter of PGTs in different tissues (**B**). Different letters indicate significant differences (*p* < 0.05). Module–PGT density correlations and corresponding *p*-values (**C**). Module–PGT size correlations and corresponding *p*-values (**D**).

In the present study, we observed the density and size of peltate glandular trichomes (PGTs) in different tissues of *O. vulgare* ‘HS’ and found that SEs were the most abundant in PGTs (≈30/mm^2^); BRs, PEs, and LEs contained a medium amount of PGTs (≈8–10/mm^2^); and STs contained the least amount of PGTs (≈2–3/mm^2^) (Figure 6A, Figure 1). Moreover, we measured the sizes of PGTs based on the diameter of the head cell and discovered that SEs showed the largest size of PGTs (≈98.68 μm), followed by BRs (≈93.02 μm), LEs (≈88.73 μm), PEs (≈87.89 μm), and STs (≈81.65 μm) (Figure 6B, Figure 1). To evaluate the association between module eigengenes and PGT density/size, we obtained correlation coefficients ranging from −0.52 to 0.95 and from −0.72 to 0.88 for PGT density and PGT size, respectively (Figure 6C, Figure 6D). Significantly, we found that the orangered4 (R = 0.95), plum1 (R = 0.67), and ivory (R = 0.61) modules showed positive correlations with PGT density, which were also obviously positive with carvacrol (Figure 5B, Figure 5C). The orangered4 (R = 0.88), lightcyan (R = 0.68), and plum1 (R = 0.51) modules showed positive correlations with PGT size (Figure 6D). Conversely, the skyblue3 module showed negative correlations with PGT density (R = −0.52) and PGT size (R = −0.62), and the darkgrey module showed negative correlations with PGT size (R = −0.72) (Figure 6C, Figure 6D).

### 2.7. WGCNA Reveals Gene Co-Expression Networks Associated with Carvacrol

**Figure 7:**
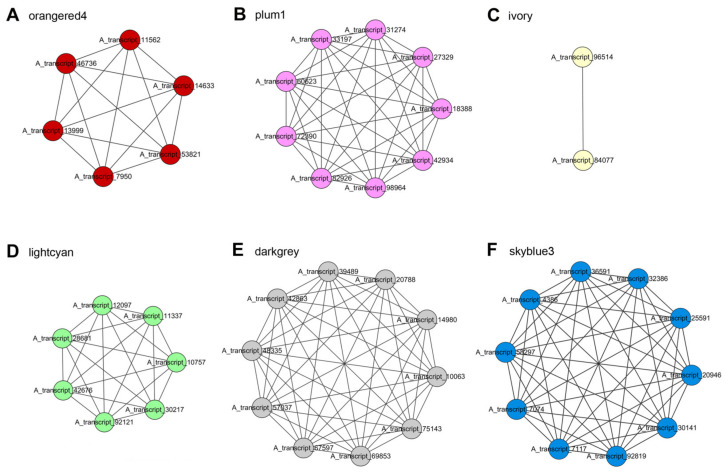
Co-expression networks of orangered4 (**A**), plum1 (**B**), ivory (**C**), lightcyan (**D**), darkgrey (**E**), and skyblue3 (**F**) constructed using carvacrol-related and peltate glandular trichome-related module genes in *Origanum vulgare* ‘Hot and Spicy’.

To evaluate the co-expression pattern of carvacrol biosynthesis-related genes in *O. vulgare* ‘HS’, we constructed networks using ‘upstream’ pathways of MVA/MEP genes and carvacrol biosynthesis genes of various TPS/CYPs and transcription factors (TFs) located in carvacrol-related or PGT-related orangered4, plum1, ivory, lightcyan, darkgrey, and skyblue3 modules (Figure 7). The co-expression network of the orangered4 module with the strongest positive correlations to carvacrol (R = 0.94) and PGT density (R = 0.95) / size (R = 0.88) contained two TPS genes, two CYP genes, one *GPPS*, and one *PDR* (Figure 7A; Table S6). The co-expression network of the plum1 module with positive correlations to carvacrol (R = 0.73) and PGT density (R = 0.64) / size (R = 0.51) contained nine genes of one *HDS* and eight TF genes, including three *bHLHs*, two *ZIPs*, one *MYB*, one *NAC*, and one *AP2/ERF-ERF* (Figure 7B; Table S6). Two co-expressed hub genes of *bHLH* and *AP2/ERF-ERF* were presented in the ivory module, which exhibited positive correlations with carvacrol (R = 0.73) (Figure 7C; Table S6). The co-expression network of lightcyan module showing positive correlations with PGT size (R = 0.68) contained seven main TF genes: four *ZIPs*, two *MYBs*, and one *WRKY* (Figure 7D; Table S6). In total, the high amount of metabolic pathway genes of TPS/CYP genes and TF genes of *ZIPs* and *bHLHs* in carvacrol-specific modules (positive correlation) suggested their significant positive roles in regulating carvacrol biosynthesis.

With negative correlations of PGT size (R = −0.72), the darkgrey module co-expression network contained one *FPPS*, one *HMGS*, five *bHLHs*, one *ZIP*, one *WRKY*, and one *AP2/ERF-ERF*, indicating the negative effects on regulating PGT development, especially for *bHLHs* (Figure 7E; Table S6). Another co-expression network of the skyblue3 module showing negative correlations with PGT density (R = −0.52) and PGT size (R = −0.62) contained ten TF genes, namely five *bHLHs*, three *WRKYs*, one *MYB*, and one *ZIP*, implying their negative roles in regulating PGT development, especially for *bHLHs* (Figure 7F; Table S6). Two TPS genes, two CYP genes, six *ZIPs*, and four *bHLHs* mainly appeared in modules positively correlated with carvacrol and PGT, suggesting their significant roles in regulating carvacrol biosynthesis and PGT development. Conversely, ten *bHLHs* and four *WRKYs* mainly appeared in modules negatively correlated with PGT, indicating their negative roles in regulating PGT development.

## 3. Discussion

### 3.1. Volatile Organic Compound Profiles in Different Tissues of O. vulgare ‘HS’

The most volatile monoterpenes found in *Origanum* are p-cymene, thymol, carvacrol, and their precursor γ-terpinene. The biosynthetic precursors of γ-terpinene and p-cymene not only determine the taste and smell of the plant, but also its properties, such as antioxidant and antimicrobial activities [18]. The aim of the present study was to use *O. vulgare* ‘HS’ as a source of carvacrol (with a very high content), which can fight against human pathogenic bacteria and fungi, as well as foodborne microbial pathogens. The accumulation of terpenoids presented tissue-specific patterns in flowers, leaves, and stems of *Origanum* species [19]. In *O. vulgare* ‘HS’, carvacrol was the main component presenting in PEs (94.40 ± 1.23%), SEs (96.92 ± 0.85%), BRs (96.07 ± 0.67%), LEs (84.71 ± 1.59%), whereas the main components in STs were p-cymene (65.44 ± 5.77%) and carvacrol (13.06 ± 6.74%) (Table S1). Other monoterpenes and sesquiterpenes (sabinene, β-caryophyllene, etc.) had lower levels in *O. vulgare* ‘HS’, as consistent with VOC composition identified in leaves of *O. vulgare* L [20]. Our study shows that *O. vulgare* ‘HS’ is a carvacrol-rich species whose VOCs exhibit tissue dependence.

### 3.2. Combined Sequencing Approach for the Different Tissues of O. vulgare ‘HS’

The understanding of structural and functional genomics is essential to comprehending plant metabolism. However, the transcripts produced from short-read sequencing may contain misassembled reads transcribed from highly repetitive areas or members of multi-gene families with extremely similar sequences [21]. PacBio RS SMRT sequencing creates a sequencing platform of the third generation with a greater error rate (up to 15%) [22]. To achieve the complete full-length transcriptome of *O. vulgare* ‘HS’, two experiments were undertaken, using the short-read sequencing and SMRT sequencing platforms. The investigation of specialized metabolite biosynthesis greatly depends on synthetic biology techniques employing genes codon-optimized for recombinant expression, which necessitates accurate and full-length cDNAs and can be hindered by inaccurate sequences, such as those predicted from genome sequences [23]. The combined sequencing approach offered us 53,802 non-redundant full-length sequences, with the N50 of 2232 bp corrected by short-read sequencing reads and CCS reads, providing a more complete view of the *O. vulgare* ‘HS’ transcriptome (Figure S3). This approach has been widely applied to other plant species, such as *Salvia miltiorrhiza* and *Drynaria roosii* [15,24].

### 3.3. Transcriptomic Insights into the Tissue-Specific Mva/Mep Pathways in O. vulgare ‘HS’

All terpenoids are derived from two universal precursors of IPP and its isomer DMAPP, which are biosynthesized in plants via two independent MEP and MVA pathways [20]. In this study, STs presented more highly expressed MVA genes than other tissues and most MEP pathway genes presented higher expression levels in SEs and BRs, followed by LEs and PEs, than in STs in *O. vulgare* ‘HS’ plants. These ‘upstream pathway’ genes presented the tissue-specific expression patterns; of these, several vital genes, such as *IDI*, *AACT*, *HMGR*, *HDR*, and *HDS* family genes, were predicted to play significant roles in generating the metabolic products of carvacrol and p-cymene. Supported by previous research, the IDI activity is of critical importance in the MVA and MEP pathways, and ensures an optimal IPP/DMAPP ratio for terpenoid precursors and/or to fuel export from plastids to cytosol [25]. The HMGRs were regarded as the rate-limiting enzymes regulating the products of mevalonate [25]. The HDRs convert HMBPP into a combination of both IPP and DMAPP, with a stoichiometry of about 5:1 [26]. Tissue transcriptomic analysis revealed higher MEP gene expression in carvacrol-rich tissues, which suggests their significant involvement in carvacrol biosynthesis.

### 3.4. Tissue-Specific Expression Patterns of TPS/CYP and SDR Genes in O. vulgare ‘HS’

The primary drivers of terpene diversification are TPSs (which generate scaffold diversity) and CYPs (which modify and further diversify these scaffolds), paving the way for further downstream modifications [17]. In the present study, we identified 62 full-length TPS genes in *O. vulgare* ‘HS’. Based on VOC contents, we found that the most carvacrol-rich tissue, SEs, presented 17 highly expressed TPS genes, including three γ-terpinene synthase genes which give rise to two different pathways yielding “cymyl” and “sabinyl” compounds, resulting in diverse chemotypes. γ-terpinene is synthesized by γ-terpinene synthase in the ‘cymyl’ pathway, which is considered to be the major precursor of carvacrol [27]. Notably, the expression patterns of three γ-terpinene synthase genes in SEs (high expression) and STs (low expression) were exactly consistent with carvacrol contents in these tissues. In both eudicots and monocots, TPS genes were predominantly found in combination with CYP71 clan genes belonging to the CYP71 family [17]. According to these results, CYP71 family genes, especially *CYP71As* and *CYP71Ds*, preferred higher transcripts in carvacrol-rich tissues of *O. vulgare* ‘HS’. Previous research showed that most monoterpenes are derived from γ-terpinene via the upregulation of *CYP71D180* and *CYP71D181*, and revealed significant correlations between the relative expression of *Ovtps7*, *Ovtps5*, and *CYP71D180* and carvacrol contents in oregano [19]. In detail, γ-terpinene was firstly oxidized by cytochrome P450 monooxygenases of the CYP71D subfamily to produce unstable cyclohexadienol intermediates. Then, the intermediates were dehydrogenated by a SDR into the corresponding ketones. The subsequent formation of the aromatic compounds occurs via keto-enol tautomerisms [10]. Similarities exist between the carvacrol biosynthesis process and the menthol biosynthesis pathway in *Mentha* × *piperita*, in which the CYP71D enzyme catalyzes the allylic hydroxylation of limonene at C-3 to form trans-isopiperitenol. Then, the SDR catalyzes the trans-isopiperitenol into the respective ketone [10,28,29]. In the current study, SDR genes also preferred the tissue-specific expression patterns of *O. vulgare* ‘HS’. SDRs are also known to contribute to the production of iridoids and cardenolides, among other terpenoids [30]. These studies demonstrate that biosynthetic routes leading to derivatives of oxygenated monoterpenes are frequently followed by ketone production via dehydrogenation and hydroxylations.

### 3.5. WGCNA of Genes Modules Related to Carvacrol Biosynthesis

The systems technique for data mining using WGCNA was exceptionally efficient in discovering information about modules shared by subsets of samples and connected to phenotypic characteristics; it is extremely adept at exposing trait-specific modules and important genes for biology sciences [31]. In this work, we used 16,341 DEGs derived from different tissues of *O. vulgare* ‘HS’ and clustered nine modules based on expression profiles. Frequently, the amount of specialized metabolites generated by a plant is strongly associated with the density of glandular trichomes present on the surface of the epidermis, and the higher EO content in oregano may be attributable to the significantly greater abundance of glandular trichomes [12,13]. Therefore, we not only observed gene modules positively correlated with carvacrol metabolite (orangered4, plum1, ivory), but also focused on the PGT-related gene modules of orangered4, plum1, and lightcyan (positive correlations) and darkgrey and skyblue3 (nagative correlations). Moreover, SEs had the highest amount of PGTs, followed by BRs, PEs, LEs, and STs in *O. vulgare* ‘HS’, which agreed with the VOC content trend in tissues, especially for carvacrol. Different *Solanum* species accumulate different contents of terpenoids due to the shape and metabolic capacity of glandular trichomes [32,33]. Measurements of PGT sizes in some Lamiaceae plants with leaf surface view were made through the diameter of the head along with the above subcuticular space [34,35]. Thus, we measured the diameter of PGTs and found that SEs showed the biggest size of PGTs, followed by BRs, LEs, PEs, and STs, similar to tissue-dependent VOC contents. Our work confirms that the development of PGTs is highly positively correlated with carvacrol biosynthesis with tissue-dependence in *O. vulgare* ‘HS’.

Progresses have been made in confirming six TF families related to the regulation of terpenoid metabolism, such as AP2/ERFs, bHLHs, MYBs, NACs, WRKYs, and bZIPs [36]. Our WGCNA results indicated that modules positively correlated with carvacrol and PGT density/size contained *ZIPs*, *bHLHs*, *MYBs*, *WRKYs*, *NACs*, and *AP2/ERF-ERFs*, and negative modules presented *bHLHs*, *WRKYs*, *ZIPs*, and *AP2/ERF-ERFs*. For instance, *CitAP2.10* and *CitERF71* regulates valencene and (E)-geraniol biosynthesis in citrus, *MsMYB* inhibits monoterpene synthesis in spearmint, and *GaWRKY1* upregulates sesquiterpene phytoalexin accumulation in cotton [37,38,39,40]. The mutation in the *NAC* binding region of TPS gene promoters affects monoterpene contents in two kiwifruit species, while overexpression of *OsbZIP79* reduces phytoalexin accumulation in rice [41,42]. As reported, a set of TFs act as positive regulators of trichome formation, and a set of TFs act as negative regulators [43]. The WGCNA modules exhibited positive or negative correlations with PGT density/size, which implies positive or negative roles in regulating PGT development, which in turn affects carvacrol biosynthesis in *O. vulgare* ‘HS’.

## 4. Materials and Methods

### 4.1. Plant Materials and RNA Sample Preparation

The *O. vulgare* ‘HS’ plants were planted in the experimental farm at the Institute of Botany of the Chinese Academy of Sciences. Five different tissues (STs, LEs, BRs, SEs, and PEs) from *O. vulgare* ‘HS’ plants at the full-bloom stage were collected and immediately frozen in liquid nitrogen, with three plant replicates per tissue. Total RNA was obtained using an RNAprep Pure Plant kit (TIANGEN Biotech, Beijing, China) according to manufacturer’s instructions. The quality of RNA was determined using a NanoDrop 2000 spectrophotometer (Thermo Fisher Scientific, MA, USA) and an Agilent 2100 bioanalyzer (Agilent Technologies, CA, USA).

### 4.2. The SPME-GC/MS Technology

The VOCs were analyzed using the SPME technique on an Agilent 7890A/7000C (Agilent Technologies, CA, USA) equipped with an Agilent HP-5MS Column (30 m × 250 μm × 0.25 μm). Supelco SPME 50/30 µm DVB/CAR/PDMS fiber was used. The SPME GC-MS cycle included 600 s pre-incubation, 80 °C incubation on the SPME GC agitator unit, and 30 min for extraction (fiber exposure) and desorption, with an oven program of 40 °C for 3 min followed by a continued increase in temperature step by step (5 °C per min to 60 °C; 2 °C per min to 88 °C; 5 °C per min to 108 °C; 2 °C per min to 140 °C; 15 °C per min to 280 °C). The helium flow rate was 2.25 mL min^−1^ and injection was split with a ratio of 200:1. The ionization potential of the mass selective detector was 70 eV and the mass range was 30–500 u. The components were identified by comparison of NIST 17 library spectra, standards (carvacrol and thymol), and retention indices (RI) with previous literature [44]. The relative percentage of individual VOCs was computed from the GC peak areas.

### 4.3. Pacbio Library and Illumina Library Construction and Sequencing

One high-quality purified RNA sample mixed from the five different tissues of STs, LEs, BRs, SEs, and PEs in *O. vulgare* ‘HS’ plants were used for PacBio library construction and sequencing. Normalized cDNAs of different sizes were constructed separately for two SMRT cell libraries using the SMARTer TM PCR cDNA Synthesis Kit. The complexes of templates and polymerase were bound to magnetic beads and then transferred to a 96-well PCR plate for processing using a PacBio Squel II system (PacBio, CA, USA). The sub-reads were filtered and subjected to CCS through Isoseq3. Fifteen individual RNA sequences of STs, LEs, BRs, SEs, and PEs were used for Illumina library construction and sequencing. The cDNA libraries for HiSeq X Ten sequencing were constructed according to a previously reported method [15]. The cDNA libraries were sequenced using Illumina Novaseq 6000 (Biomarker Technologies, Beijing, China).

### 4.4. Transcript Annotation and Differential Expression Analysis

The annotation information was obtained via mapping non-redundant transcripts to NR, Swissprot, GO, COG, KOG, Pfam, and KEGG databases. The short-read sequencing data was mapped to SMRT data to achieve quantitative analysis without assembly. In order to ensure the quality of gene expression analysis, our short-read sequencing data were subjected to a quality evaluation including the Fragments Per Kilobase of transcript per million fragments mapped (FPKM) density distribution, expression quantity of samples, and expression relevance analysis between samples. According to the correlation results, DEGs were obtained on the condition of false discovery rate (FDR) < 0.01 and an absolute value of |log2 fold change (FC)| ≥ 1 based on the DESeq method [45].

### 4.5. Gene Co-expression Network Analysis and Network Visualization

Co-expression and module analyses were performed using functions of the R package WGCNA (V1.47) [31]. The soft threshold Pearson correlation was applied to construct a gene expression adjacency matrix between all pairs in the probe expression dataset. A soft power threshold of 1 was applied to transform the correlation matrix into a signed weighted adjacency matrix, producing an approximate scale-free topology for the given network (R^2^ = 0.8). The fine modules were clustered using a dynamic hybrid tree-cut algorithm (R package dynamic Tree Cut, v. 1.62) and DeepSplit was set to 2. Modules with highly similar eigengenes (dissimilarity, 0.25) were merged to avoid oversplitting clusters. The network visualization was conducted via Cytoscape_v.3.0.0 (https://cytoscape.org/).

### 4.6. Statistical Analysis

Analysis of variance was performed to compare the significant differences using SPSS software (version 25.0, IBM, NY, USA). A supervised statistical data method was run using PLS-DA with SIMCA software (Version 14.1; Umetrics, Umea, Sweden).

## 5. Conclusions

In summary, VOCs varied a lot in different tissues of aerial parts of *O. vulgare* ‘HS’, with carvacrol representing a large percentage of all VOCs. The molecular mechanisms of carvacrol biosynthesis were therefore investigated using metabolomics and transcriptomics approaches. First of all, the combination of SMRT sequencing and short-read sequencing was used to achieve complete full-length transcripts, which provided the basic genetic information of *O. vulgare* ‘HS’. Based on the gene expression and metabolite profiles, modular organization analysis was conducted to obtain the metabolite-related gene modules. Considering the involvement of PGTs in carvacrol accumulation, WGCNA results illuminated the crosstalk between significant metabolic pathway genes and relevant TF genes distributed in carvacrol- and PGT-correlated modules, which indicates their positive or negative roles in regulating carvacrol biosynthesis in *O. vulgare* ‘HS’.

## Data Availability

The SMRT sequencing and HiSeq X Ten data were submitted to the National Center for Biotechnology Information (NCBI) under accession number PRJNA700511.

## Supplementary Materials

The following supporting information can be downloaded at https://www.mdpi.com/article/10.3390/ijms232113231/s1.
