# Supplementary material for: Unraveling the Biosynthesis of Carvacrol in Different Tissues of Origanum vulgare
Source: Int J Mol Sci. 2022 Oct 30;23(21):13231. doi: 10.3390/ijms232113231 (PMC9654750; doi:10.3390/ijms232113231)
Supplement: Supplementary file 1 [file ijms-23-13231-s001.zip › ijms-1930249-supplementary.pdf]

International Journal of
Molecular Sciences

MDPI

Appendix

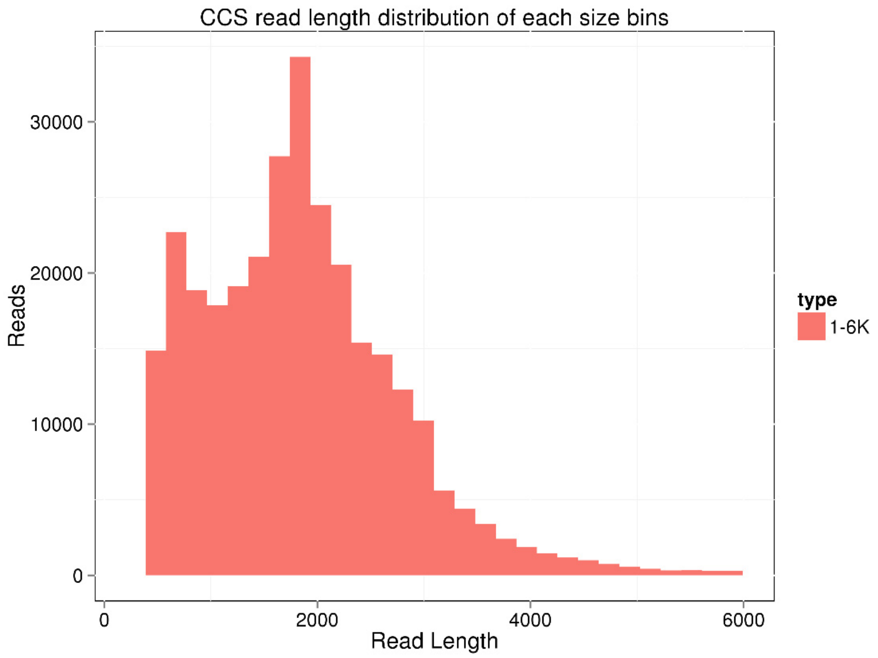

Figure S1. Circular consensus read length distribution of *Origanum vulgare* 'Hot & Spicy'.

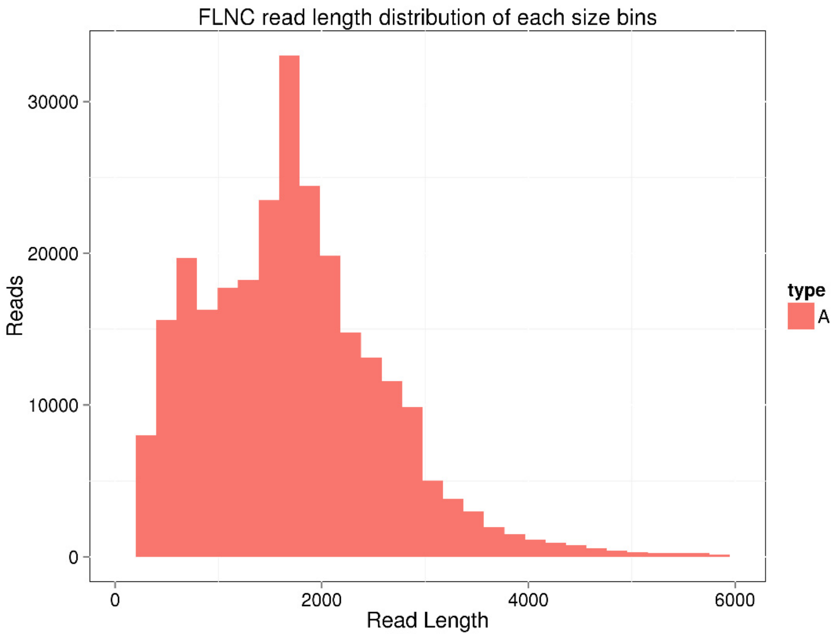

Figure S2. Full-length non-chimeric read length distribution of *Origanum vulgare* 'Hot & Spicy'.

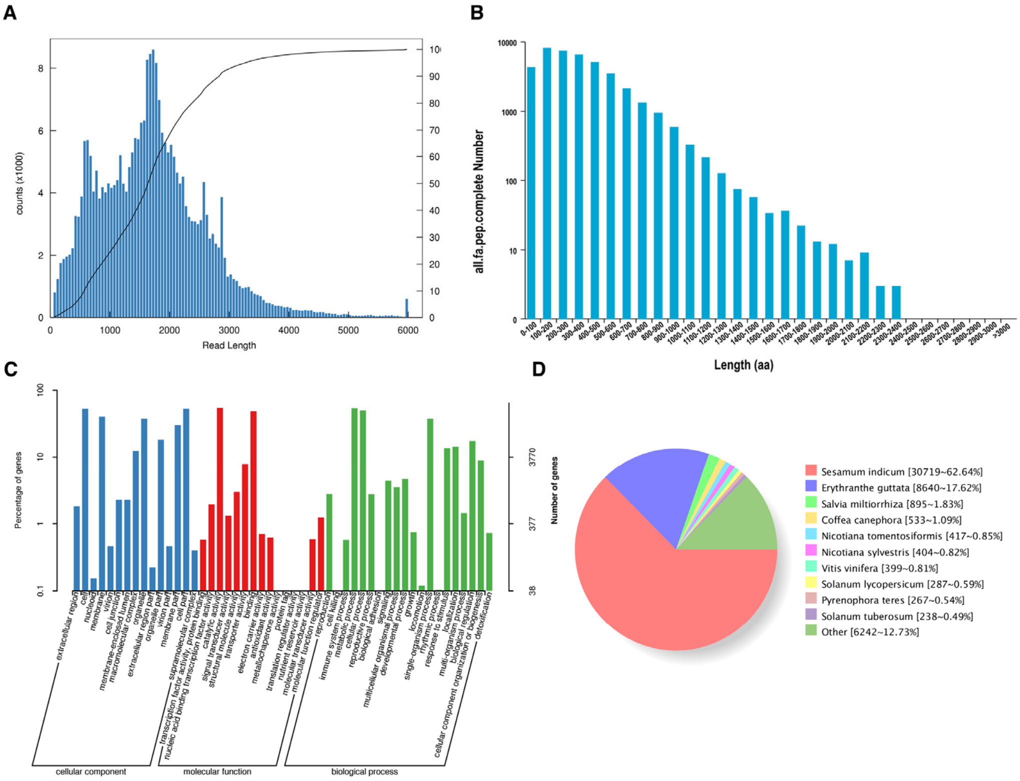

Figure S3. The full-length transcriptome sequencing and assembly of *Origanum vulgare* 'Hot & Spicy' using the SMRT method. (A) Length distribution of full length non-chimeric (FLNC) sequences. (B) Length distribution of predicated protein sequences. (C) Functional annotation of FLNC sequences based on GO categorization. The main functional categories within the biological process, cellular component and molecular function categories are relevant to plant physiology. The right y-axis indicates the number of FLNC sequences. The left y-axis indicates the percentage of FLNC sequences. (D) NR classification diagram.

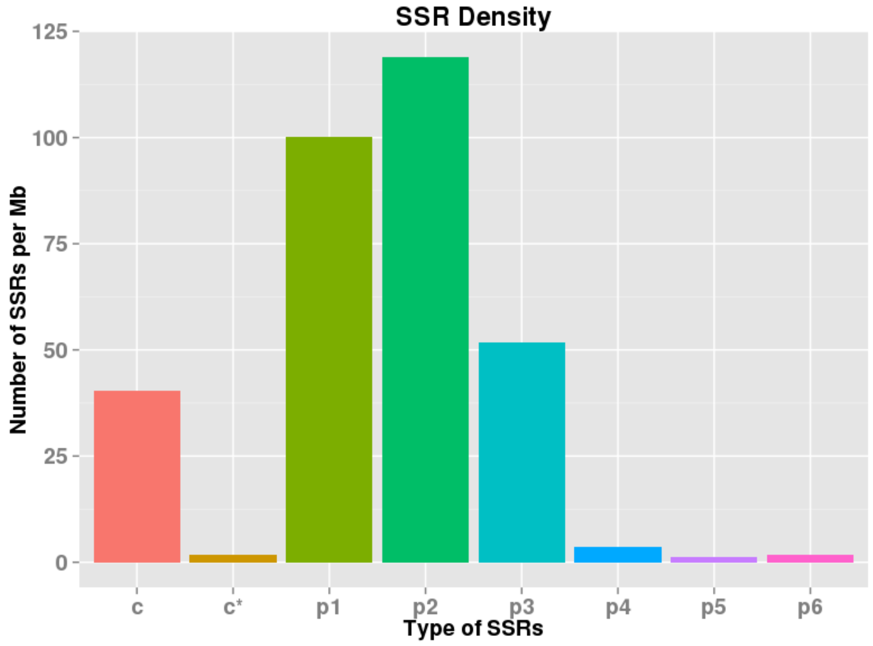

Figure S4. SSR density distribution of *Origanum vulgare* 'Hot & Spicy'.

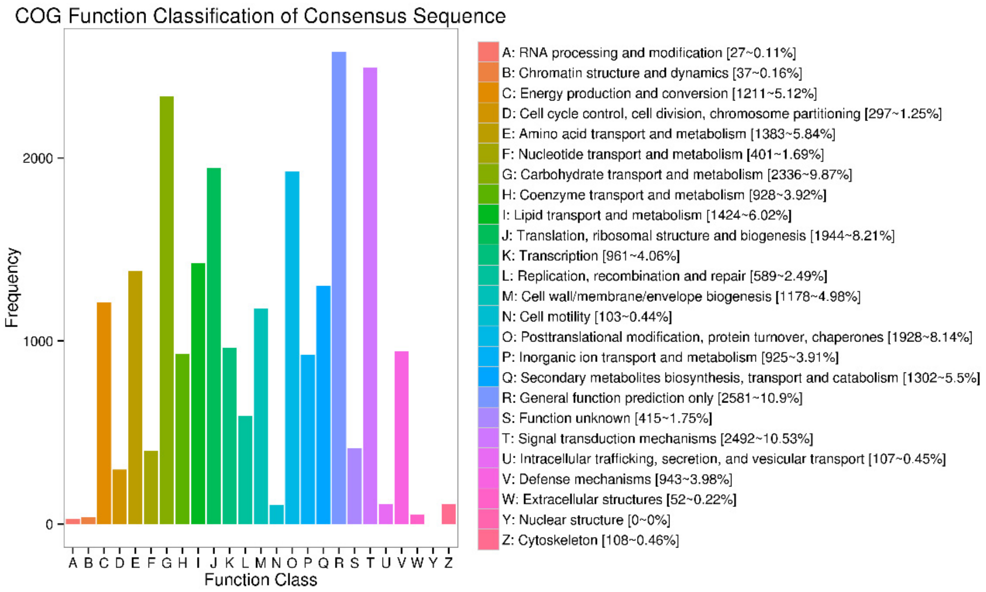

Figure S5. COG function classification of consensus sequences of *Origanum vulgare* 'Hot & Spicy'.

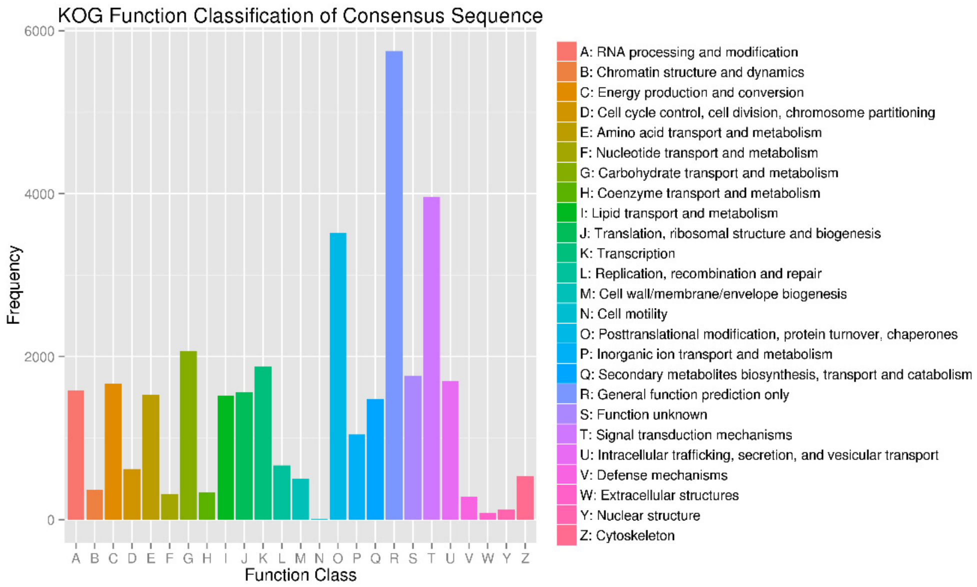

Figure S6. KOG function classification of consensus sequences of *Origanum vulgare* 'Hot & Spicy'.

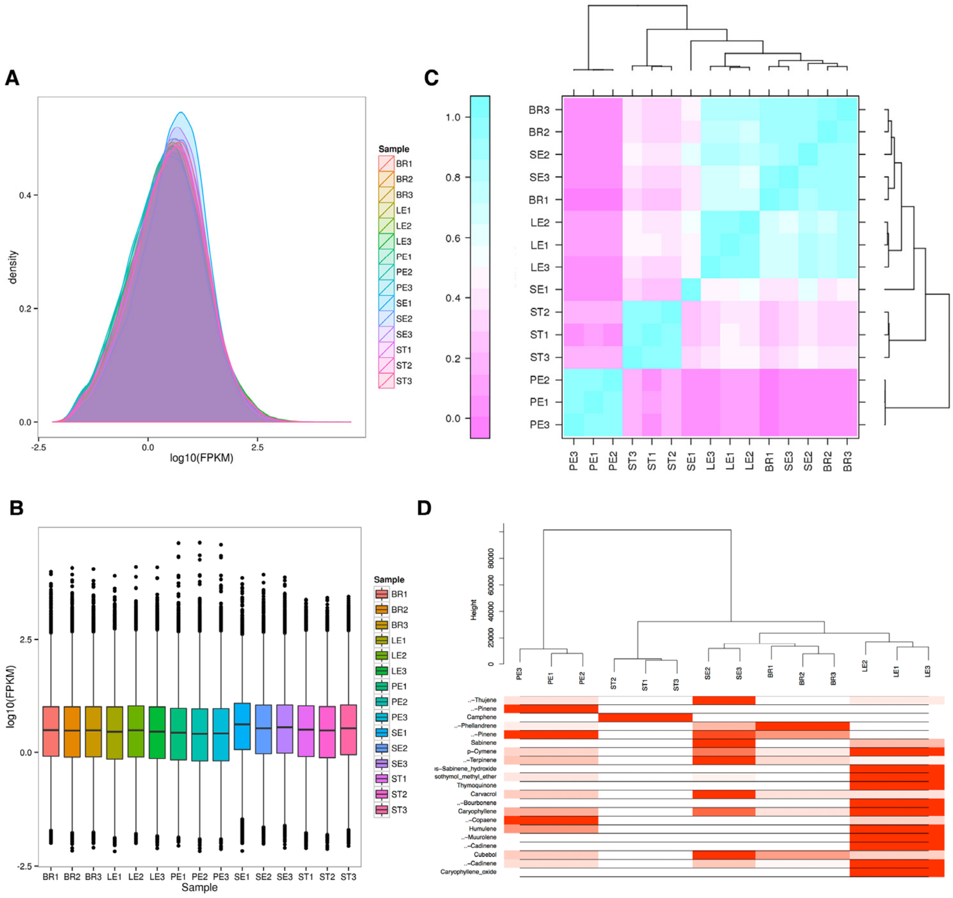

Figure S7. Next generation sequencing data analysis of *Origanum vulgare* 'Hot & Spicy' using stem (ST; ST1-3), leaf (LE; LE1-3), bract (BR; BR1-3), sepal (SE; SE1-3) and petal (PE; PE1-3)] samples. (A) Comparison diagram of FPKM density distribution. (B) Boxplot of FPKM average values. (C) Pearson Correlation Coefficient analysis of expression levels. (D) Pearson Correlation Coefficient.

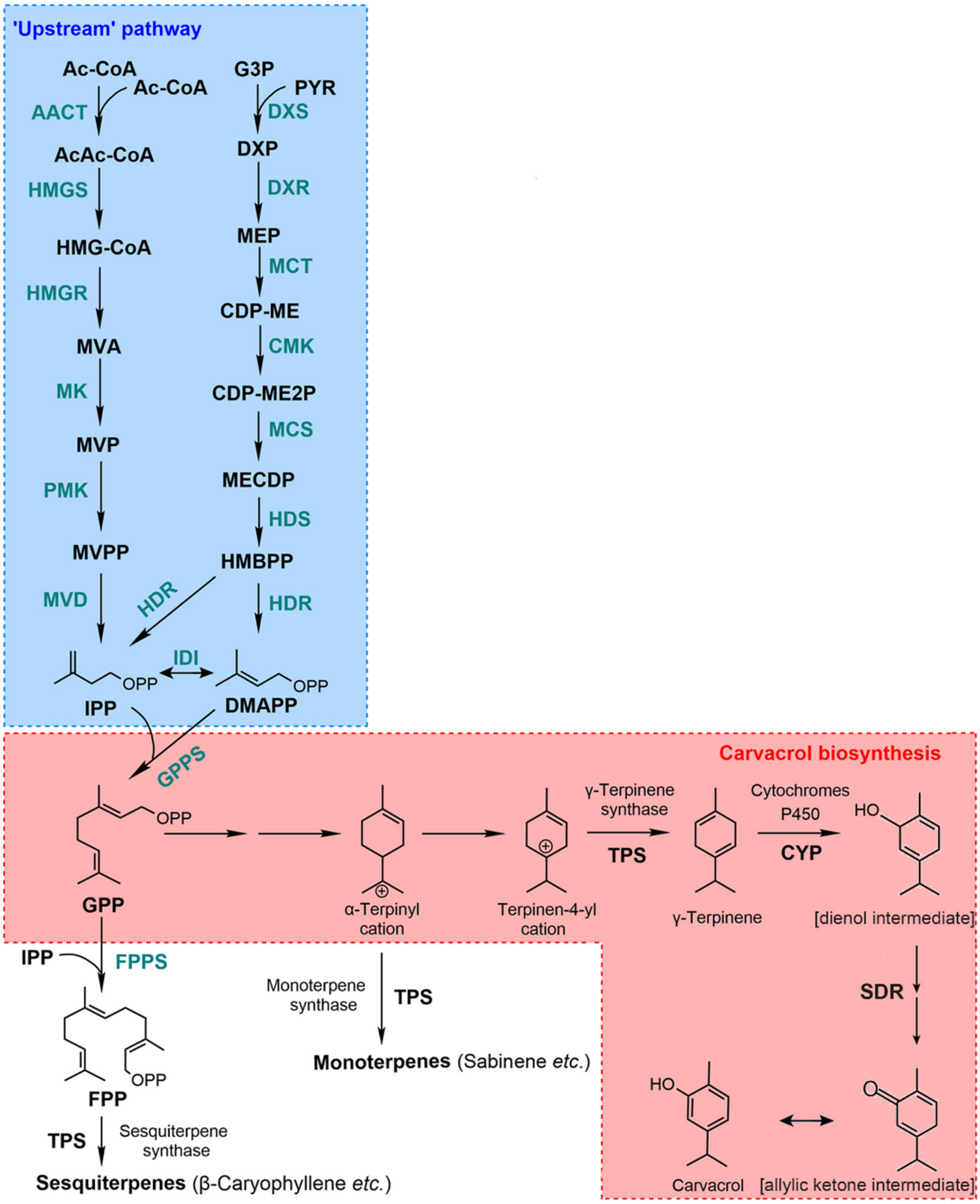

Figure S8. Putative schematic representation of the monoterpene/sesquiterpene biosynthetic pathways in *O. vulgare* 'HS'. The main pathway refers to the carvacrol biosynthesis, which origins from 'upstream' pathways of MVA and MEP pathways. Abbreviations: AACT, acetoacetyl-CoA thiolase; Ac-CoA, acetyl-coenzyme A; AcAc-CoA, acetoacetyl-coenzyme A; CMK, CDP-ME kinase; CDP-ME, 4-diphosphocytidyl-2-C- methyl-D-erythritol; CDP-ME2P, 4-diphosphocytidyl-2-C-methyl-D-erythritol 2-phosphate; CYP, Cytochromes P450, DMAPP, dimethylallyl diphosphate; DXP, 1-deoxy-D-xylulose-5-phosphate; DXR, DXP reductoisomerase; DXS, DXP synthase; FPP; farnesyl diphosphate; FPPS, FPP synthase; G3P, glyceraldehyde 3-phosphate; GPP; geranyl diphosphate; GPPS, GPP synthase; HDR, HMBPP reductase; HDS, HMBPP synthase; HMBPP, 1-hydroxy-2-methyl-2-(E)-butenyl 4-diphosphate; HMG-CoA, hydroxymethylglutaryl-coenzyme A; HMGR, HMG-CoA reductase; HMGS, HMG-CoA synthase; IDI, isopentenyl diphosphate isomerase; IPP, isopentenyl diphosphate; MCS, MECDP synthase; MCT, MEP cytidylyltransferase; MECDP, 2- C-methyl-D-erythritol 2,4-cyclodiphosphate; MEP, 2-C-methyl-D-erythritol-4-phosphate; MK, mevalonate kinase; MVA, mevalonate; MVD, mevalonate diphosphate decarboxylase; MVP, 5-phosphomevalonate; MVPP, 5-diphosphomevalonate; PMK; phosphomevalonate kinase; PYR, pyruvate; TPS, terpene synthase.

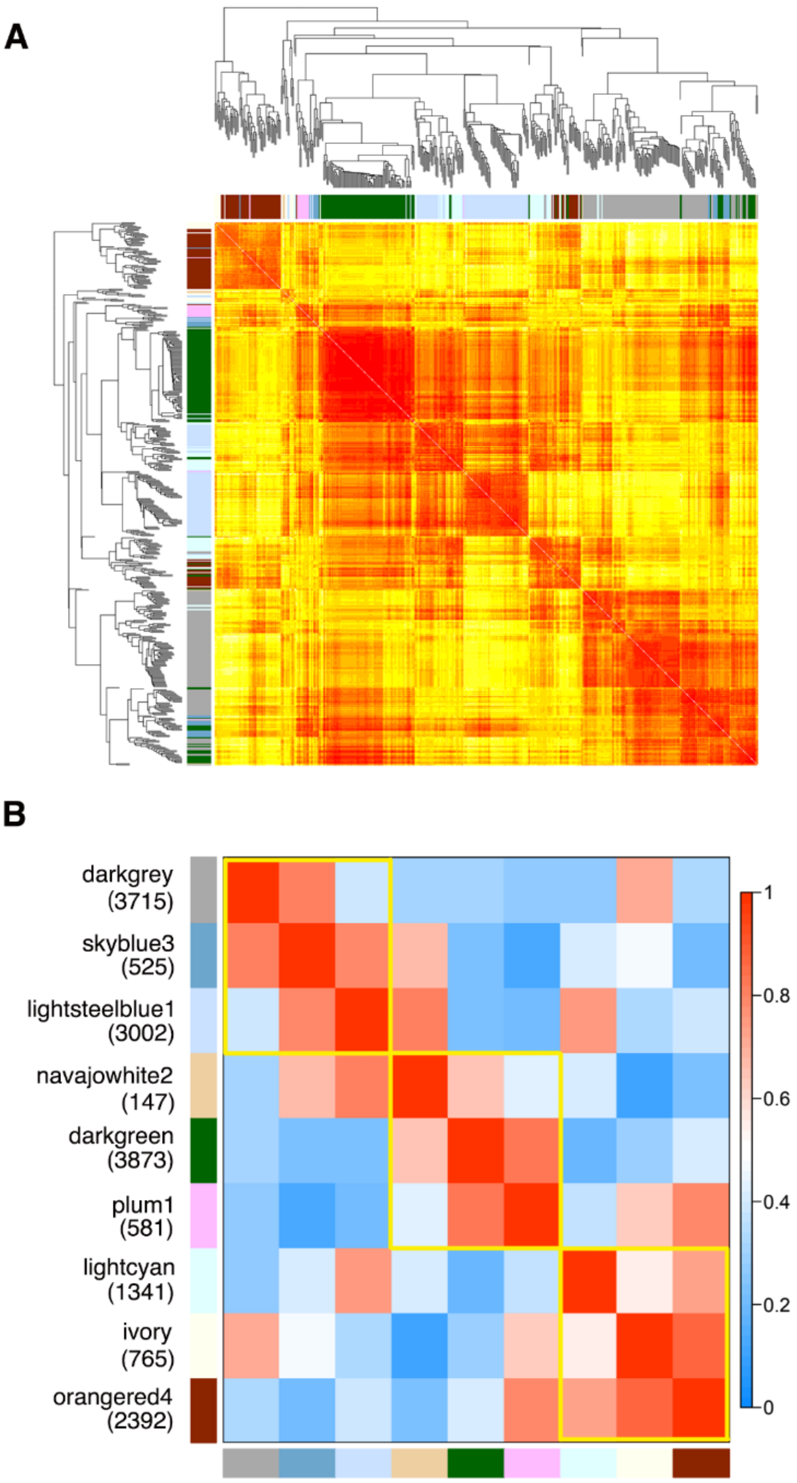

Figure S9. Identification of co-expression network modules in *Origanum vulgare* 'Hot & Spicy'. (A) Network heat map plot. Branches in the hierarchical clustering dendrograms correspond to modules. Color-coded module membership is indicated by the colored bars below and to the right of the dendrograms. In the heat map, high co-expression interconnectedness is indicated by progressively more saturated yellow and red colors. Modules correspond to blocks of highly interconnected genes. Genes with high intramodular connectivity are located at the tip of the module branches because they display the highest interconnectedness with the rest of the genes in the module. (B) Module eigengene adjacency heat map. The heat map shows the relatedness of the nine co-expression modules identified in WGCNA, with red indicating highly related and blue indicating not related.

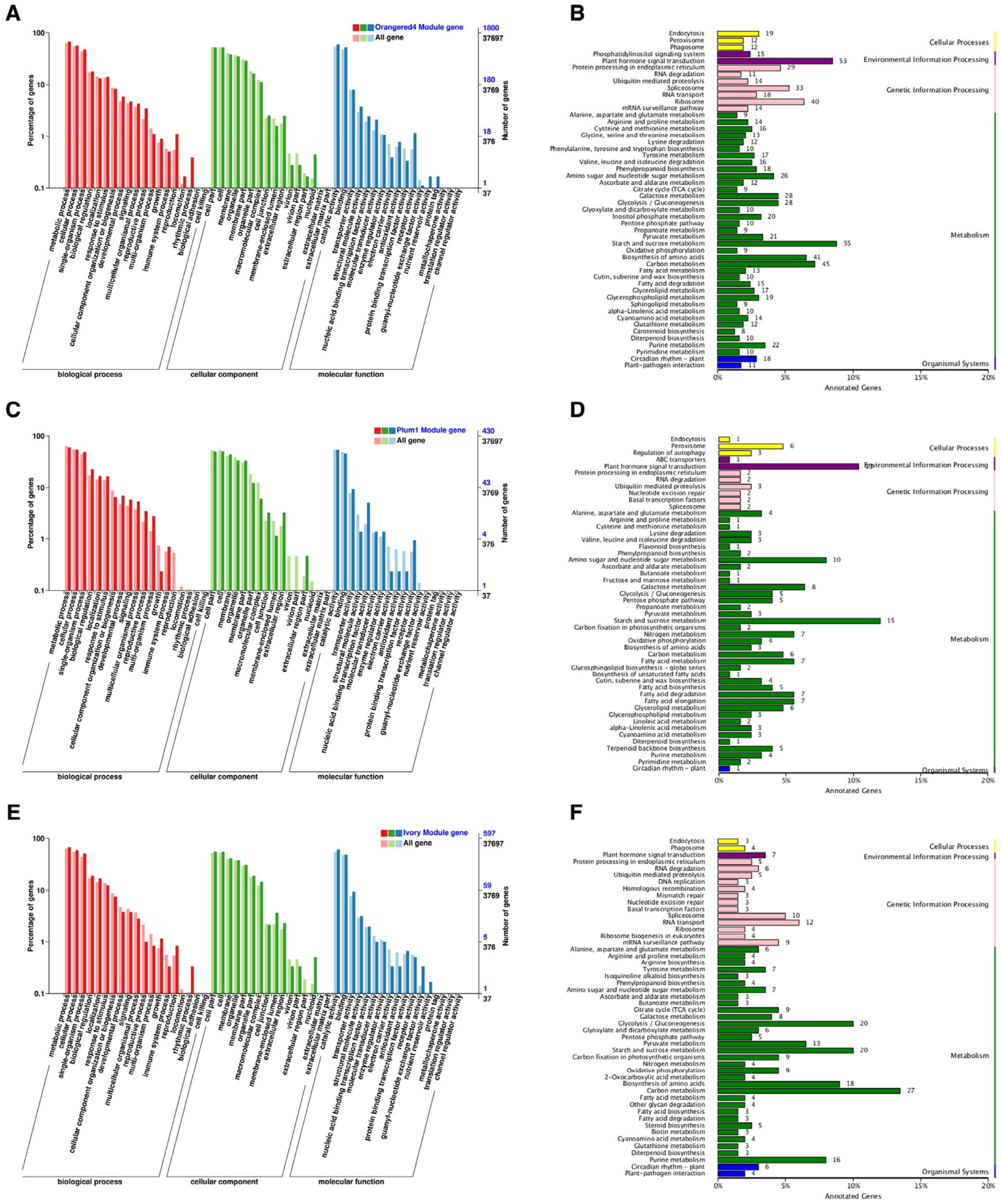

Figure S10. Enrichment analysis of GO terms and KEGG terms in *Origanum vulgare* 'Hot & Spicy'. Functional annotation of genes based on GO categorization (A,C,E) and KEGG categorization (B,D,F) in orangered4, plum1 and ivory modules positive to carvacrol content. The bars represent the percentage and numbers of genes with BLASTX matches to each GO and KEGG term.

Table S1. Relative percentage of all VOCs in different tissues of *Origanum vulgare* 'Hot & Spicy' via SPME-GC/MS.

Compound	RI	Relative percentage of all volatile compounds (%)				
		Stem	Leaf	Bract	Sepal	Petal
α-Thujene	923	-	0.23±0.20	-	0.36±0.12	0.23±0.2
α-Pinene	928	0.74±0.64	-	-	-	0.06±0.05
Camphene	942	0.60±0.52	-	-	-	-
β-Pinene	970	0.16±0.14	-	0.11±0.11	0.03±0.03	0.14±0.13
Sabinene	971	-	0.09±0.03	-	0.05±0.01	-
α-Phellandrene	992	0.94±0.17	-	0.37±0.33	0.02±0.02	0.02±0.02
p-Cymene	1022	65.44±5.77	5.58±0.82	0.81±0.80	0.67±0.28	0.91±0.24
γ-Terpinene	1056	-	0.28±0.31	0.81±0.15	0.68±0.20	0.82±0.14
cis-Sabinene hydroxide	1065	-	0.05±0.06	-	-	-
Carvacrol methyl ether	1245	8.46±1.52	0.70±0.16	-	0.01±0.01	0.07±0.01
Thymoquinone	1249	-	0.96±0.82	-	-	-
Carvacrol	1302	13.06±6.74	84.71±1.59	96.07±0.67	96.92±0.85	94.40±1.23
β-Bourbonene	1379	0.57±0.07	0.32±0.04	-	-	-
β-Caryophyllene	1413	2.64±0.50	3.33±0.55	0.54±0.48	0.44±0.39	0.95±0.85
β-Copaene	1432	0.22±0.19	0.26±0.09	-	0.17±0.15	0.84±0.14
Humulene	1446	-	0.32±0.12	-	-	0.12±0.02
α-Muurolene	1495	1.13±0.27	0.33±0.14	-	-	-
γ-Cadinene	1509	0.63±0.01	0.44±0.08	-	-	-
Cubebol	1509	0.27±0.08	0.05±0.03	0.12±0.02	0.04±0.03	0.04±0.00
δ-Cadinene	1521	0.93±0.14	0.80±0.27	0.04±0.04	0.04±0.04	0.10±0.02
Caryophyllene oxide	1582	1.71±0.30	0.16±0.15	-	-	-
Total		97.5±0.68	98.6±0.43	98.87±0.51	99.44±0.42	98.68±1.27

Note: Values represent averages of 3 determinations; retention index (RI) relative to standard mixture of n-alkanes on HP-5MS column.

Table S2. Length distribution of predicted proteins of *Origanum vulgare* 'Hot & Spicy'.

Protein length span	Numbers	Percent (%)
0~100	4243	10.51
100~200	8028	19.89
200~300	7294	18.07
300~400	6444	15.96
400~500	5017	12.43
500~600	3478	8.62
600~700	2094	5.19
700~800	1313	3.25
800~900	938	2.32
900~1000	583	1.44
1000~1100	324	0.8
1100~1200	213	0.53
1200~1300	125	0.31
1300~1400	74	0.18
1400~1500	56	0.14
1500~1600	33	0.08
1600~1700	36	0.09
1700~1800	22	0.05
1800~1900	13	0.03
1900~2000	12	0.03
2000~2100	7	0.02
2100~2200	9	0.02
2200~2300	3	0.01
2300~2400	3	0.01
2400~2500	0	0
2500~2600	1	0
2600~2700	1	0
2700~2800	1	0
2800~2900	0	0
2900~3000	0	0
3000~3100	0	0

Table S3. Statistical table of NGS data evaluation of *Origanum vulgare* 'Hot & Spicy'.

Samples	Read number	Base number	GC content (%)	Q30 (%)
ST1	22,059,888	6,595,387,786	49.52	93.96
ST2	22,917,197	6,852,978,628	49.57	94.14
ST3	22,148,162	6,622,477,418	49.14	93.07
LE1	22,461,638	6,726,138,800	49.63	93.89
LE2	21,644,784	6,478,706,764	49.45	93.09
LE3	22,104,584	6,609,676,930	49.93	93.54
BR1	21,804,366	6,525,817,980	49.28	93.82
BR2	22,183,732	6,632,682,292	49.43	93.77
BR3	21,531,867	6,435,083,368	49.81	93.51
SE1	25,476,878	7,629,805,698	48.33	93.47
SE2	22,483,143	6,730,521,762	49.48	94.08
SE3	22,278,039	6,660,271,094	49.01	94.20
PE1	22,008,438	6,586,780,886	49.92	93.46
PE2	21,431,964	6,413,527,672	50.08	93.26
PE3	22,338,367	6,685,089,802	49.93	93.31

Table S4. Statistical results of comparations between NGS-based data and SMRT-based non-redundant transcript data of *Origanum vulgare* 'Hot & Spicy'.

Sample name	Total reads	Uniquely mapped reads (%)	Percent of reads mapped to multiple loci (%)	Percent of reads mapped to too many loci (%)
ST1	22,059,888	35.12	45.03	2.75
ST2	22,917,197	35.27	45.35	2.65
ST3	22,148,162	35.13	44.36	2.63
LE1	22,461,638	34.88	46.13	3.99
LE2	21,644,784	35.27	45.29	4.00
LE3	22,104,584	34.93	45.59	4.15
BR1	21,804,366	34.02	45.08	3.96
BR2	22,183,732	32.68	46.45	5.24
BR3	21,531,867	32.34	47.39	5.12
SE1	25,476,878	36.75	43.06	2.31
SE2	22,483,143	34.92	43.96	3.30
SE3	22,278,039	34.32	43.87	3.02
PE1	22,008,438	31.99	51.39	2.46
PE2	21,431,964	32.47	51.34	2.13
PE3	22,338,367	32.26	51.00	2.27

Table S5. Statistical table of the number of DEGs.

Compare pairs	Number of DEGs	Number of up-regulated DEGs	Number of down-regulated DEGs
ST *vs* BR	10,051	4,623	5,428
ST *vs* LE	10,951	5,758	5,193
ST *vs* PE	14,762	7,596	7,166
ST *vs* SE	10,378	4,940	5,438
LE *vs* BR	4,662	1,712	2,950
LE *vs* PE	14,930	7,339	7,591
LE *vs* SE	9,455	4,759	4,696
BR *vs* PE	10,572	5,982	4,590
BR *vs* SE	1,718	1,437	281
SE *vs* PE	9,091	4,845	4,246

Table S6. Overview of co-expression network genes in orangered4, plum1, ivory, lightcyan, darkgrey and skyblue3 modules of *Origanum vulgare* 'Hot & Spicy' associated with carvacrol and PGT.

Correlation	Network module	Gene ID	Gene abbreviation
positive with carvacrol/PGT	orangered4	A_transcript_53821	PDR
positive with carvacrol/PGT	orangered4	A_transcript_46736	GPPS
positive with carvacrol/PGT	orangered4	A_transcript_14633	CYP
positive with carvacrol/PGT	orangered4	A_transcript_13999	CYP
positive with carvacrol/PGT	orangered4	A_transcript_11562	TPS
positive with carvacrol/PGT	orangered4	A_transcript_7950	TPS
positive with carvacrol/PGT	plum1	A_transcript_18388	bHLH
positive with carvacrol/PGT	plum1	A_transcript_27329	ZIP
positive with carvacrol/PGT	plum1	A_transcript_31274	AP2/ERF-ERF
positive with carvacrol/PGT	plum1	A_transcript_33197	bHLH
positive with carvacrol/PGT	plum1	A_transcript_42934	HDS
positive with carvacrol/PGT	plum1	A_transcript_60623	ZIP
positive with carvacrol/PGT	plum1	A_transcript_72390	MYB
positive with carvacrol/PGT	plum1	A_transcript_82926	bHLH
positive with carvacrol/PGT	plum1	A_transcript_98964	NAC
positive with carvacrol	ivory	A_transcript_96514	AP2/ERF-ERF
positive with carvacrol	ivory	A_transcript_84077	bHLH
positive with PGT	lightcyan	A_transcript_10757	ZIP
positive with PGT	lightcyan	A_transcript_11337	ZIP
positive with PGT	lightcyan	A_transcript_12097	ZIP
positive with PGT	lightcyan	A_transcript_28681	MYB
positive with PGT	lightcyan	A_transcript_30217	ZIP
positive with PGT	lightcyan	A_transcript_42676	WRKY
positive with PGT	lightcyan	A_transcript_92121	MYB
negative with PGT	darkgrey	A_transcript_69853	FPPS
negative with PGT	darkgrey	A_transcript_67597	bHLH
negative with PGT	darkgrey	A_transcript_57037	HMGS
negative with PGT	darkgrey	A_transcript_48335	ZIP
negative with PGT	darkgrey	A_transcript_42863	bHLH
negative with PGT	darkgrey	A_transcript_39489	bHLH
negative with PGT	darkgrey	A_transcript_20788	WRKY
negative with PGT	darkgrey	A_transcript_14980	bHLH
negative with PGT	darkgrey	A_transcript_100633	AP2/ERF-ERF
negative with PGT	darkgrey	A_transcript_75143	bHLH
negative with PGT	skyblue3	A_transcript_20946	MYB
negative with PGT	skyblue3	A_transcript_25591	WRKY
negative with PGT	skyblue3	A_transcript_30141	bHLH
negative with PGT	skyblue3	A_transcript_32386	ZIP
negative with PGT	skyblue3	A_transcript_36591	bHLH
negative with PGT	skyblue3	A_transcript_4386	bHLH

negative with PGT	skyblue3	A_transcript_58297	WRKY
negative with PGT	skyblue3	A_transcript_7074	bHLH
negative with PGT	skyblue3	A_transcript_7117	bHLH
negative with PGT	skyblue3	A_transcript_92819	WRKY
